# The Role of BAFF-R Signaling in the Growth of Primary Central Nervous System Lymphoma

**DOI:** 10.3389/fonc.2020.00682

**Published:** 2020-05-27

**Authors:** Xiaolan Zhou, Matthias Mulazzani, Iven-Alex von Mücke-Heim, Sigrid Langer, Wenlong Zhang, Hellen Ishikawa-Ankerhold, Martin Dreyling, Andreas Straube, Louisa von Baumgarten

**Affiliations:** ^1^Department of Neurology, Ludwig Maximilians University, Munich, Germany; ^2^Department of Internal Medicine I, Ludwig Maximilians University, Munich, Germany; ^3^Department of Internal Medicine III, Ludwig Maximilians University, Munich, Germany

**Keywords:** PCNSL, CRISPR-Cas9, intravital microscopy, brain tumor, knockout, BAFF-R

## Abstract

Primary CNS lymphoma (PCNSL) is an aggressive brain tumor. Despite improvements in therapeutic algorithms, long-term survival remains rare, illustrating an urgent need for novel therapeutic targets. BAFF-R is a pro-survival receptor expressed on most malignant B cells, including PCNSL. To date, its role in PCNSL growth remains elusive. Here, we have created a BAFF-R knockout lymphoma cell line (BAFF-R-KO) using CRISPR-Cas9. In serum-starved conditions, BAFF-R-KO cells exhibit decreased viability *in vitro* compared to BAFF-R^+^ cells. Combining an orthotopic mouse model of PCNSL with chronic cranial windows and intravital microscopy, we have demonstrated a significant delay in tumor growth in mice inoculated with BAFF-R-KO cells compared to BAFF-R^+^ PCNSL. Additionally, median survival of BAFF-R-KO mice was significantly prolonged. Altogether, our results indicate the high potential of BAFF-R as a novel treatment target for PCNSL.

## Introduction

B-cell activating factor (BAFF) is a key cytokine promoting B-cell maturation, proliferation, and survival (1). BAFF binds to three receptors: BAFF-Receptor (BAFF-R), transmembrane activator and CAML-interactor (TACI), and B-cell maturation antigen (BCMA) (2). While the receptors TACI and BCMA bind both BAFF and a proliferation-inducing ligand (APRIL), BAFF-R exclusively interacts with BAFF. BAFF-R is mainly expressed on B cells (3). Both BAFF-R^−/−^ and BAFF^−/−^ mice show severe reduction of mature B-cell populations (4, 5). In contrast, neither BCMA^−/−^ nor TACI^−/−^ mice exhibit B-cell deficiency (6, 7), confirming BAFF-R as the primary receptor for BAFF-mediated B-cell survival. Transgenic BAFF overexpression in lymphoid cells leads to B-cell hyperplasia and autoimmunity (8, 9). Additionally, BAFF expression is highly upregulated in several subtypes of non-Hodgkin lymphoma (NHL), with increased BAFF serum levels correlating with more aggressive disease and worse outcome (10–12). BAFF-R is expressed on many B-cell malignancies, including diffuse large B-cell lymphoma (DLBCL), and its activation increases proliferation and survival of DLBCL cells (13–17). Blockade of the BAFF pathway using a toxin fused to BAFF (rGel/BLyS fusion toxin) significantly reduced tumor growth in a DLBCL xenograft model (18).

A total of 95% of all primary central nervous system lymphomas (PCNSL) are of the DLBCL subtype, accounting for 3–5% of primary brain tumors (19). Nearly 30% of PCNSL are refractory to therapy, and up to 50% of the patients relapse (20), highlighting the urgent need for novel therapeutic strategies.

In PCNSL, BAFF transcription and expression has been shown for both malignant B cells and astrocytes (21). In line with these findings, our group has demonstrated a high expression of BAFF as well as its receptors in PCNSL specimens (22), indicating a potential role for autocrine BAFF/BAFF-R signaling in the pathophysiology of PCNSL. Patients with PCNSL showed significantly higher BAFF CSF levels when compared with other focal brain lesions (23, 24). However, patients with PCNSL also exhibit higher CSF levels of soluble TACI and soluble BCMA (25), possibly limiting the local availability of BAFF (26).

Although BAFF-R expression in PCNSL has been described before, its importance for PCNSL growth remains unclear. To address this question, we constructed a BAFF-R knockout DLBCL cell line by utilizing CRISPR/Cas9-mediated genome engineering and determined the specific contribution of BAFF-R to DLBCL proliferation and survival *in vitro* as well as *in vivo* using an orthotopic mouse model.

## Materials and Methods

### Cell Culture

U-2932 cells, a human DLBCL cell line, were cultured in Iscove's Modified Dulbecco's Medium (Life Technologies, Germany) supplemented with 20% human serum, 0.4% heparin, and 0.1% beta-mercaptoethanol. Cell cultures were regularly checked for mycoplasma infections using a PCR Mycoplasma Test Kit (PanReac AppliChem GmbH, Germany). Cell line authentication was performed using short tandem repeat profiling (DSMZ, Germany).

#### TdTomato Transfection

To enable long-term intravital microscopy, U-2932 cells were stably transfected with the red fluorescent protein tdTomato. The tdTomato plasmid (catalog no. 632531, TaKaRa Clontech, USA) was cloned into the lentiviral vector pLVX-IRES-neoR (catalog no. 632181, TaKaRa Clontech, USA) and transfected using electroporation (Gene Pulser Xcell system, Bio-Rad Laboratories, USA). Clones with sufficient tdTomato expression for intravital microscopy (U-2932-tdt) were selected by use of G418 and six iterations of FACS sorting.

#### BAFF-R Knockout Using CRISPR/Cas9

**Figure 1:**
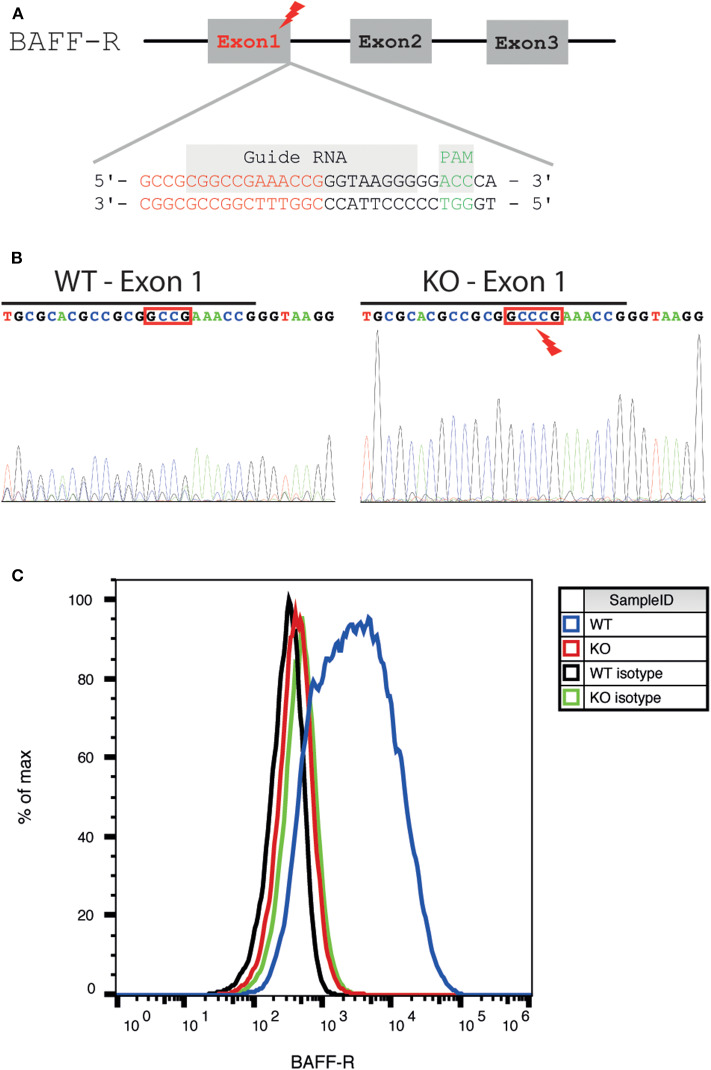
Generation and validation of a BAFF-R knockout cell line. **(A)** Schematic diagram of the location of the gRNA binding site in the BAFF-R gene. The genomic region the end of exon 1 was targeted (end of exon 1 highlighted in red, gRNA sequence framed in purple, and PAM sequence in green). **(B)** Sanger sequencing showing insertion of 1 nucleotide (C) into exon 1, causing a frameshift mutation. The end of exon 1 was marked with a black line. **(C)** Flow cytometry revealed loss of BAFF-R expression in the U-2932-tdt-BAFF-R-KO cell line.

BAFF-R KO cell lines were established using CRISPR/Cas9-mediated genome engineering. The gRNA-pSpCas9-BB-2A-GFP-PX458 plasmid was obtained from GenScript (USA): a 20 bp guide RNA (gRNA) complementary to the end of the first exon of the BAFF-R gene (sequence: CCCTTACCCGGTTTCGGCCG, Figure 1A) was designed using a dedicated software (Zhang Laboratory MIT CRISPR Design Tool, http://crispr.mit.edu). The plasmid was electroporated into U-2932-tdt. Single cells with positive GFP expression were FACS sorted using a MoFlo Astrios Cell Sorter (Beckman Coulter, Germany), transferred into one-cell-cultures and expanded.

The region including the guide sequence of exon 1 was amplified by polymerase chain reaction (PCR). Primers (sequence 5′AGGGGCAGTCCTCCGTCAAA3′ and 5′AGGGGCTGAATTGGGGAACCAC3′) were acquired from Metabion (Germany). Proof reading Platinum Pfx DNA polymerase (Invitrogen, USA) was used for high-fidelity. After knockout, PCR products were purified, and Sanger sequencing was conducted to verify gene disruption.

#### Flow Cytometry

Reagents for cell surface staining were acquired from Biolegend (San Diego, USA). To analyze membrane expression of the three BAFF receptors, lymphoma cells were preincubated with a human FcγR blocking reagent (5 μl per million cells) and incubated with APC-anti-TACI, Alexa Fluor 647 (AF647)-conjugated anti-BAFF-R, AF647-conjugated anti-BCMA, or with their corresponding isotypes for 20 min on ice (5 μl per million cells). Thereafter, the cells were washed and analyzed using a Gallios Flow Cytometer (Beckman Coulter).

#### Quantification of Cell Proliferation and Viability

To examine cell proliferation under different conditions, 2.5 × 10^4^ U-2932-tdt cells, U-2932-tdt-BAFF-R-KO cells, or U-2932-tdt cells incubated with a neutralization anti-BAFF-R antibody (20 μg/ml, AF1162, R&D Systems, USA) were cultured in a 96-well-plate in serum-free or serum-containing medium for a period of 24, 48, and 72 h. Cell proliferation was determined using the MTS assay (Promega, Germany) according to the manufacturer's instructions. Measurements represented the mean of three identical wells. All experiments were performed in triplicate.

To examine cell viability, 10^6^ U-2932-tdt cells or U-2932-tdt-BAFF-R-KO cells per well were seeded in a 6-well-plate in serum-free or serum-containing medium for a period of 24, 48, and 72 h. Cells were counted using a Neubauer counting chamber, cell viability was determined with Trypan Blue. All experiments were done in triplicate.

### Animal Model

All animal experiments were approved by the local authorities and performed in accordance with the German and Bavarian animal welfare regulations. Homozygous Foxn1^nu/nu^ mice were purchased from The Jackson Laboratory via Charles River Laboratories (Germany) and kept in our facilities in accordance with the guidelines of the Federation of European Laboratory Animal Associations (FELASA). Experiments were commenced in Foxn1^nu/nu^ mice aged 8–14 weeks. Allocation of animals to treatment group was randomized. Investigators were blinded for group allocation until completion of statistical analyses.

#### Orthotopic PCNSL Model and Survival Analysis

10^6^ U-2932-5d5 cells or U-2932-tdt-BAFF-R KO cells were stereotactically injected into the brain parenchyma in 1–2 μl DPBS after borehole craniotomy. The position was 2 mm posterior and 1.5 mm lateral to bregma in the left cranial hemisphere at a depth of 3.5 mm from the bone surface. Mice were euthanized if symptoms of predefined severity occurred or weight loss exceeded 20%.

#### Microsurgical Cranial Window Implantation

Cranial window implantation was performed as described (27–29). After anesthesia (intraperitoneal injection of medetomidine, midazolam, and fentanyl) and subcutaneous injection of cefotaxime and dexamethasone, mice were placed on a heating pad (37°C) and fixed in a modified surgery frame (David Kopf Instruments, USA). A circular piece of skull (5.5 mm diameter) and the dura mater were carefully removed. Then, a transparent, sterile cover glass (6 mm diameter) was attached to the skull using dental glue (Cyano veneer, Hager Werken, Germany). After 2 weeks of recovery, the cover glass was removed, and 2.5 × 10^5^ U-2932-tdt cells or U-2932-tdt-BAFF-R-KO cells in 1–2 μl DPBS were stereotactically injected into the mouse cortex using a 10 μL microsyringe and a 32-gauge needle (Hamilton, USA). Cells were injected 2 mm posterior of bregma and 1 mm lateral of the sagittal sinus at an intracortical depth of 1 mm. A custom-made polyether ether ketone ring (8 mm diameter) was attached to the skull to enable fixation for intravital microscopy.

#### Repeated Intravital Fluorescence Microscopy

To monitor tumor growth over time, an Olympus microscope with a 4x objective (numerical aperture 0.28, Olympus XLFluor 4x/340) and a CCD camera (Hamamatsu, France) were used. During imaging, mice were anesthetized with 1–2% isoflurane in oxygen and immobilized in a custom-made fixation device. Imaging was conducted at days 14, 21, 28, and 35 after stereotactic tumor implantation.

#### Analysis of Tumor Growth

The acquired tdTomato fluorescence images were analyzed with ImageJ/Fiji (National Institutes of Health, USA). To create a mosaic, the automatic alignment function of the Grid/Collection Stitching plugin was used. For tumor area measurement, the outline of the tumor was manually delineated.

#### Immunofluorescence and Tumor Volume Measurement

Intracardiac perfusion with DPBS, followed by 4% paraformaldehyde solution, was performed 35 days after tumor implantation. Mouse brains were extracted, frozen, and stored at −80°C. For sectioning, 15 μm cryosections spaced 495 μm apart were cut using a cryostat (Leica CM 1950, Leica, Germany). For immunofluorescence staining, goat anti-human CD20 (3 μg/ml, ab194970, abcam, United Kingdom) was used as a primary antibody, and donkey AF594-conjugated anti-goat antibody (1:200, A11058, Thermo Fisher Scientific, USA) was used as a secondary antibody. DAPI (Sigma-Aldrich, USA) was used for nuclear staining. Slices were observed with a BX60 upright microscope (Olympus, Japan). Tumor area was manually delineated on each section using Axiovision software (Carl Zeiss Microscopy). Total tumor area per slice was multiplied with 0.495 mm, and addition of all values yielded total tumor volume.

### Statistical Analysis

Calculations were performed using Graphpad Prism Version 7.02. Results are expressed as mean ± SEM or median + interquartile range, as indicated. Mann-Whitney *U*-test, repeated measures two-way ANOVA, or regular two-way ANOVA with Tukey's multiple comparisons test was used, as indicated. Survival was plotted using the Kaplan-Meier method, and differences were analyzed using the Gehan-Breslow-Wilcoxon test. A *p* < 0.05 was considered statistically significant.

## Results

### Generation of a Stable BAFF-R Knockout Lymphoma Cell Line

To investigate the effect of BAFF-R expression in PCNSL, we generated a DLBCL lymphoma cell line lacking BAFF-R via CRISPR/Cas9-mediated knockout targeting exon 1 (Figure 1A). After successful BAFF-R knockout in the U-2932 cell line (U-2932-tdt-BAFF-R-KO), Sanger sequencing of a single-cell clone revealed an insertion of cytosine leading to a frame shift mutation in the BAFF-R gene (*TNFRSF13C*; Figure 1B). FACS analysis confirmed the absence of this transmembrane receptor (Figure 1C). Theoretically, knockout of BAFF-R may lead to increased expression of the other two known BAFF receptors, BCMA and TACI. However, compensatory upregulation of BCMA or TACI expression was ruled out using FACS analysis (Supplemental Figures 1A,B). Taken together, we were able to successfully create a stable BAFF-R knockout in a DLBCL cell line.

### BAFF-R Is Crucial for Lymphoma Cell Survival and Proliferation *in vitro*

**Figure 2:**
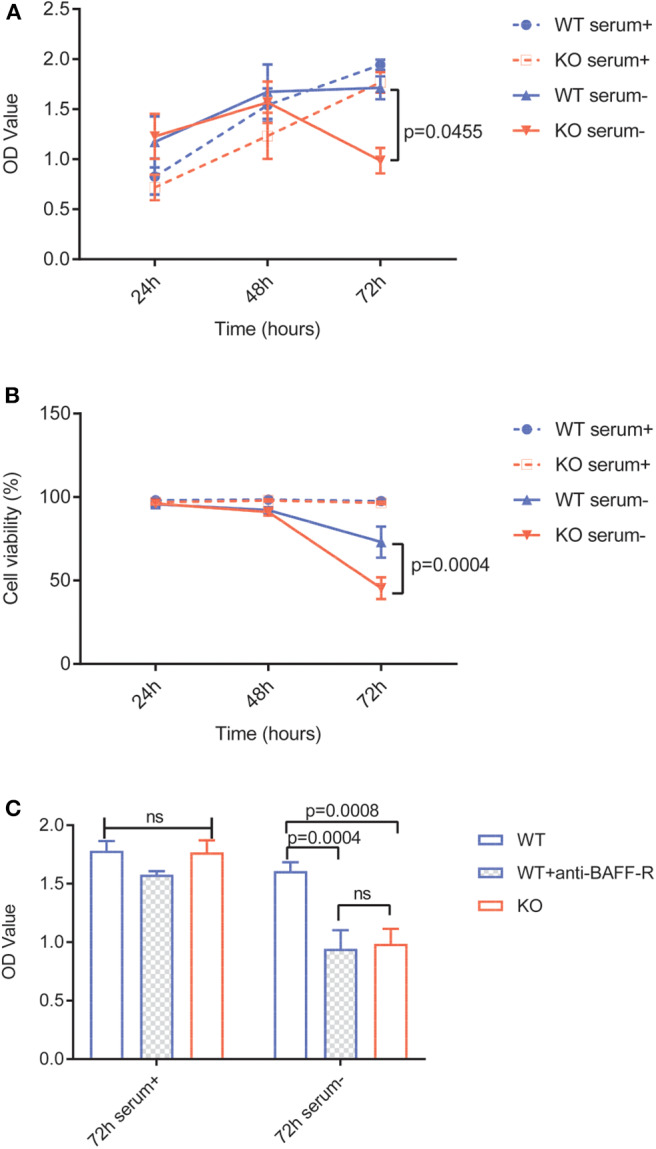
BAFF-R knockout and treatment with an anti-BAFF-R-antibody impedes cell survival and proliferation in serum-free medium. **(A)** MTS assay showing U-2932-tdt (WT) and U-2932-tdt-BAFF-R-KO (KO) proliferation in serum-containing or serum-free medium. Data are shown as mean ± SEM (*n* = 3). Two-way repeated measures ANOVA with Tukey's adjustment for multiple comparisons and BAFF-R KO without serum vs. BAFF-R WT without serum at 72 h after incubation, *p* = 0.0455. **(B)** Trypan blue assay showing viability of U-2932-tdt (WT) and U-2932-tdt-BAFF-R-KO cells in serum-containing or serum-free medium. Data are shown as mean ± SEM (*n* = 3). Two-way repeated measures ANOVA with Tukey's adjustment for multiple comparisons and BAFF-R KO without serum vs. BAFF-R WT without serum at 72 h after incubation, *p* = 0.00004. **(C)** MTS assay showing proliferation of U-2932-tdt (WT) cells, U-2932-tdt (WT) cells incubated with BAFF-R antibody (20 μg/ml), and U-2932-tdt-BAFF-R-KO (KO) cells in serum-containing or serum-free medium after 72 h. Data are shown as mean ± SEM (*n* = 3). Two-way ANOVA with Tukey's adjustment for multiple comparisons.

To investigate the specific role of BAFF-R expression in lymphoma cells, we evaluated cell viability and proliferation *in vitro*. Due to a plethora of different growth-stimulating factors present in human serum, we performed the experiments in both serum-containing and serum-free media. In the presence of human serum, viability and growth of U-2932-tdt-BAFF-R-KO cells showed no difference to U-2932-tdt cells (Figures 2A,B). In serum-free medium, however, U-2932-tdt-BAFF-R-KO cells exhibited reduced viability and reduced proliferation compared to U-2932-tdt cells after 72 h in culture (Figures 2A,B). To confirm the dependence of cellular proliferation on BAFF-R expression, we performed another experiment in the presence and absence of the neutralizing BAFF-R antibody AF1162 (Figure 2C). As expected, addition of a BAFF-R neutralizing antibody led to significantly reduced proliferation of U2932-tdt cells, similar to the reduction seen in U-2932-tdt-BAFF-R-KO cells. Taken together, these results confirm that in serum-free medium, BAFF-R is essential for sustained lymphoma cell survival and proliferation *in vitro*.

### BAFF-R Knockout Leads to Reduced Tumor Growth and Prolonged Animal Survival in an Orthotopic Model of PCNSL

**Figure 3:**
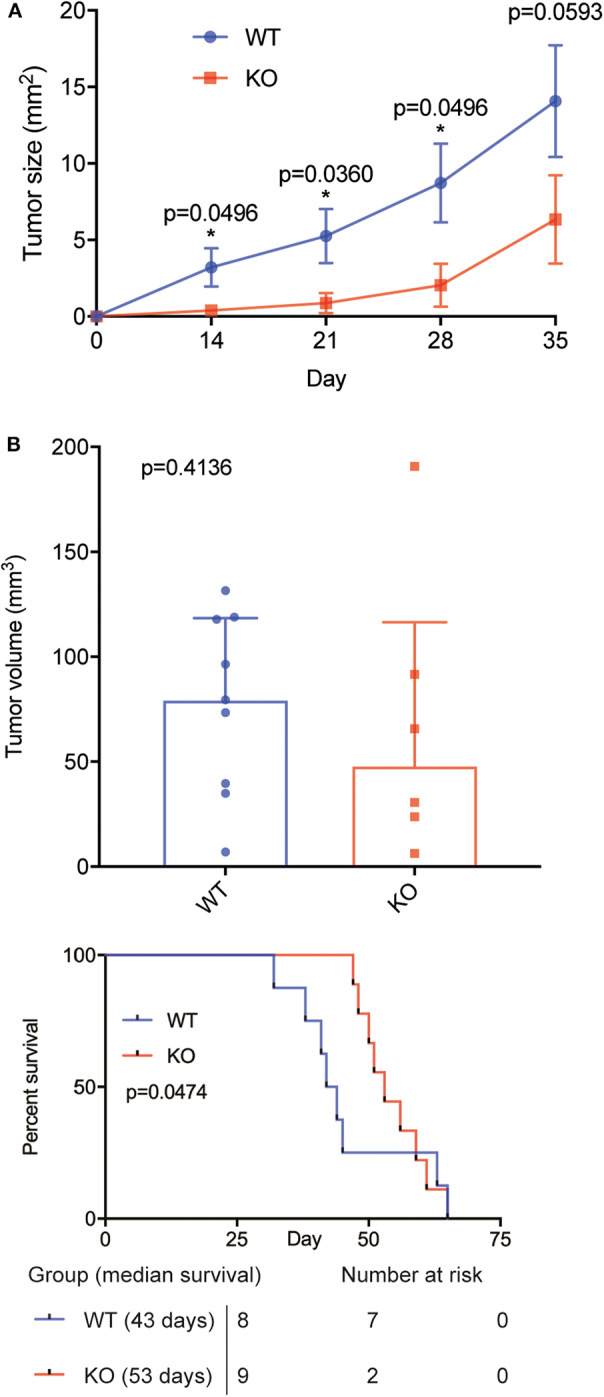
BAFF-R knockout slows tumor growth and prolongs animal survival in an orthotopic model of PCSNL. **(A)** Longitudinal evaluation of tumor area using *in vivo* microscopy, *n* = 9 mice until day 28, *n* = 8 mice on day 35 (U-2932-tdt), and *n* = 6 mice (U-2932-tdt-KO). Data are shown as mean ± SEM, *p*-values shown for Mann Whitney U tests. Median values WT vs. KO: 2.23 vs. 0.05 (day 14), 4.37 vs. 0.18 (day 21), 9.15 vs. 0.56 (day 28), and 16.48 vs. 3.02 (day 35). **(B)** Tumor volume measured by immunofluorescence at day 35, *n* = 8 (U-2932-tdt) and *n* = 6 (U-2932-tdt-BAFF-R-KO). Mann-Whitney *U*-test, *p* = 0.4136. Data shown as median + interquartile range. **(C)** Kaplan–Meier survival plot of mice after stereotactic implantation of U-2932-tdt or U-2932-tdt-BAFF-R-KO cells, *n* = 8 and *n* = 9, respectively, median survival 43 days vs. 53 days, Gehan-Breslow-Wilcoxon test, *p* = 0.0474.

Next, we sought to validate our findings in an orthotopic model of PCNSL (29). Microsurgical implantation of a chronic cranial window enabled us to longitudinally monitor intracranial tumor growth *in vivo* using epifluorescence microscopy. Knockout of BAFF-R led to reduced tumor growth, which was significant on day 14, 21 and 28 (Figure 3A, Supplemental Figure 2). On day 35, however, the difference did not reach significance (*p* = 0.059). Immunofluorescence analysis of tumor size 35 days after tumor implantation showed smaller mean tumor volume in the knockout group (KO: mean 68.12, range 6.30–190.76. WT: mean 77.44, range 6.94–131.55), although this difference was not statistically significant (*p* = 0.41) (Figure 3B).

Survival analysis showed that knockout of BAFF-R significantly prolonged median survival (Figure 3C; 53 days (range: 47–65) compared to 43 days (range: 32–65); *p* = 0.0474). Taken together, knockout of BAFF-R slows tumor growth during the first 4 weeks after tumor implantation and significantly prolongs median survival.

## Discussion

BAFF-R is a transmembrane protein upregulated in many B-cell malignancies, including DLBCL cells. In this study, we confirmed the specific contribution of BAFF/BAFF-R signaling for lymphoma cell survival and proliferation *in vitro* and established its importance for PCNSL growth *in vivo*.

Our *in vitro* results confirm previous reports highlighting the role of the BAFF/BAFF-R axis in malignant B cells' resistance to spontaneous or drug-induced apoptosis (16, 17, 30). BAFF-R signaling induces pro-survival effects in malignant B cells via NF-κB activation (17) and by functioning as a transcriptional regulator (31). Similar to our results, inhibition of BAFF-R does not affect the viability of malignant B cells when cultured in serum-containing medium, illustrating the compensation by other survival factors in serum-containing medium (17). However, we observed that, in an *in vitro* environment devoid of other survival factors, autocrine BAFF/BAFF-R signaling plays a crucial role in DLBCL survival and proliferation. Similarly, murine BAFF-R^−/−^ pre-B acute lymphoblastic leukemia (pre-B ALL) cells had similar viability and proliferation rates to their wildtype counterparts. However, BAFF-R^−/−^ pre-B ALL cells were more sensitive to drug treatment, indicating that BAFF-R signaling potentially confers a survival advantage during treatment (30). Thus, the widespread expression and upregulation of BAFF-R in B-cell malignancies and its pro-survival signaling renders it a potential target for cancer therapy.

Our *in vivo* findings illustrate a significant growth inhibition in BAFF-R knockout cells within the first 4 weeks after tumor implantation. However, 35 days after tumor implantation this difference does not reach significance (*p* = 0.0593 for tumor area and 0.4136 for tumor volume). Animal survival, which was assessed in another cohort of animals receiving tumor cell injections into the deep brain parenchyma, showed a survival benefit for animals bearing BAFF-R KO tumors. In both models, the effect on tumor growth and animal survival seems to be more pronounced at an early stage of tumor growth.

A possible explanation for these findings may be the variable dependence on BAFF-R signaling at different stages of PCNSL growth. Initially, BAFF-R/NF-κB-mediated stimulation of cell proliferation and reduction of apoptosis may lead to faster tumor growth of BAFF-R^+^ lymphoma cells (Figure 3A). However, adaptive changes occur during consecutive tumor growth to its cellular, vascular, and metabolic microenvironment. Large immune cell infiltrates accumulate in solid tumors over time, including PCNSL (32, 33). These non-tumor cells are known to supply direct and indirect growth mediators stimulating tumor cell proliferation (34). Similarly, lymphoma cells produce autocrine and paracrine growth mediators such as IL-10 (35), IL-6, and VEGF (36), and higher lymphoma cell numbers in bigger tumors suggest higher absolute production of these cytokines at later time points. Together, the abundance of growth mediators at late stage lymphoma growth may reduce the dependence on BAFF-R signaling over time. This is also supported by our *in vitro* results showing that BAFF-R knockout did not affect growth in serum-containing media, whereas it significantly reduced the viability and proliferation rate in serum-free media.

Based on this data it is tempting to speculate that BAFF-R targeted therapy may show increased effectiveness in early stage PCNSL; however, further preclinical studies are needed to prove this hypothesis.

Monoclonal antibodies against soluble BAFF have already been approved and used successfully in the treatment of autoimmune diseases (37–40). Although their application in B-cell malignancies has not yet reached similarly satisfying results, preclinical experiments have highlighted the potential of targeting the BAFF/BAFF-R axis in CLL (41) and ALL (30). Furthermore, novel BAFF-R antibodies have recently been developed to specifically target BAFF-R on malignant B cells. These antibodies proved highly effective to specifically lyse a range of malignant B cells and to suppress the growth of various systemic B-cell malignancies, including NHL (42–44). However, further studies are needed to determine the efficacy of BAFF-R antibodies in PCNSL. In contrast to systemic NHL, PCNSL resides behind the blood–brain barrier. This complex structure is composed of several cell types connected by tight junctions, regulating the entry of cells and molecules into the brain. Antibodies are large molecules with limited penetrance across the intact blood-brain barrier. Therefore, the intratumoral accumulation of intravenously injected antibodies in PCNSL patients remains debated. Nevertheless, patients with PCNSL exhibit a disrupted blood–brain barrier, as illustrated by characteristic contrast-enhancement in CT and MRI. Furthermore, a recent meta-analysis showed that the addition of rituximab (a CD20-targeting antibody) to methotrexate-based chemotherapy may improve progression-free survival in immunocompetent patients with newly diagnosed PCNSL (45). Therefore, further preclinical studies are warranted to evaluate the effect of BAFF-R specific antibodies on PCNSL, preferentially in combination with methotrexate-based chemotherapy. Small-molecule inhibitors against BAFF-R could offer a brain-penetrating alternative to antibodies (46). Additionally, also chimeric antibody receptor-based approaches targeting BAFF-R have been described, further expanding the spectrum of treatment options targeting BAFF-R (47, 48). As BAFF-R is expressed on several peripheral B-cell subsets, however, B-cell depletion has to be expected as a potential side effect of such treatments, similar to rituximab or anti-CD19 CAR T cell treatment.

Here, we have shown that the expression of BAFF-R confers a survival advantage for malignant B cells *in vitro* and leads to accelerated PCNSL growth *in vivo*. Therefore, our data supports the preclinical evaluation of combinatory treatment protocols additionally targeting the BAFF/BAFF-R axis in PCNSL.

## Data Availability Statement

The datasets generated for this study are available on request to the corresponding author.

## Ethics Statement

The animal study was reviewed and approved by Regierung von Oberbayern, Germany.

## Author Contributions

XZ carried out the conceptualization, methodology, validation, formal analysis, investigation, writing (original draft), and visualization. MM carried out the conceptualization, methodology, formal analysis, writing (original draft), review and editing, and the visualization. I-AM-H carried out the formal analysis, and writing (original draft). SL carried out the project administration, methodology, and investigation. WZ carried out the investigation. HI-A carried out the investigation. MD carried out the management of resources. AS carried out the management of resources. LB carried out the conceptualization, methodology, validation, writing, review, editing, supervision, and funding acquisition.

## Conflict of Interest

MM has been a member of a scientific advisory committee for Gilead. MD has been a member of a scientific advisory committee for Novartis. The remaining authors declare that the research was conducted in the absence of any commercial or financial relationships that could be construed as a potential conflict of interest.

## Supplementary Material

Supplemental Figure 1 BCMA and TACI expression in U-2932-tdt-BAFF-R-KO. **(A)** Flow cytometry revealed similar BCMA expression in the U-2932-tdt-BAFF-R-KO cell line. **(B)** Flow cytometry revealed similar TACI expression in the U-2932-tdt-BAFF-R-KO cell line.

Supplemental Figure 2 Individual tumor area as measured via epifluorescence. *n* = 9 mice until day 28, and *n* = 8 mice on day 35 (U-2932-tdt, blue); *n* = 6 mice (U-2932-tdt-KO, red).
