# Supplementary figures and images for: The Role of BAFF-R Signaling in the Growth of Primary Central Nervous System Lymphoma
Source: Front Oncol. 2020 May 27;10:682. doi: 10.3389/fonc.2020.00682 (PMC7266954; doi:10.3389/fonc.2020.00682)

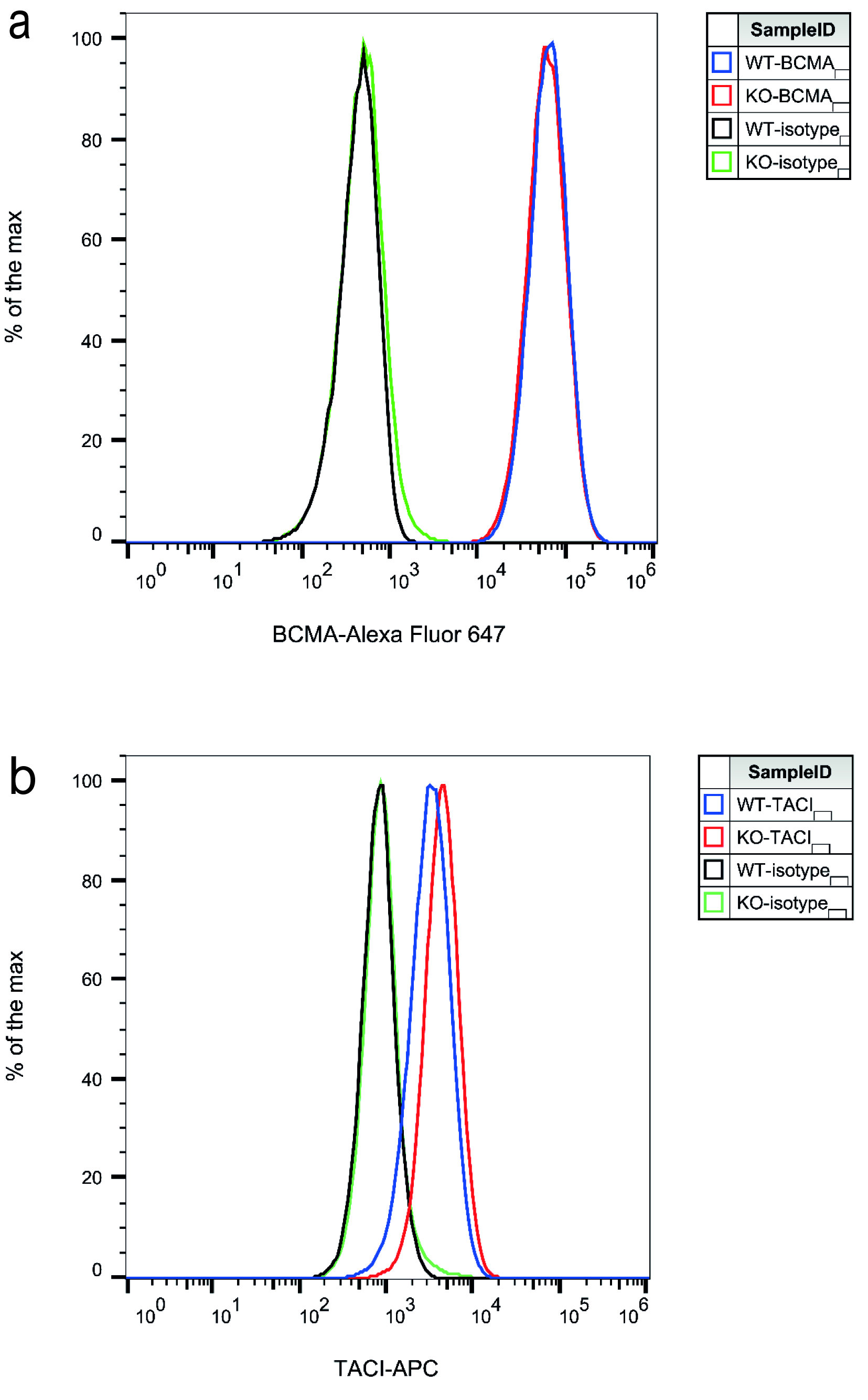

Supplement: Supplemental Figure 1 — BCMA and TACI expression in U-2932-tdt-BAFF-R-KO. (A) Flow cytometry revealed similar BCMA expression in the U-2932-tdt-BAFF-R-KO cell line. (B) Flow cytometry revealed similar TACI expression in the U-2932-tdt-BAFF-R-KO cell line. [file Image_1.TIFF]

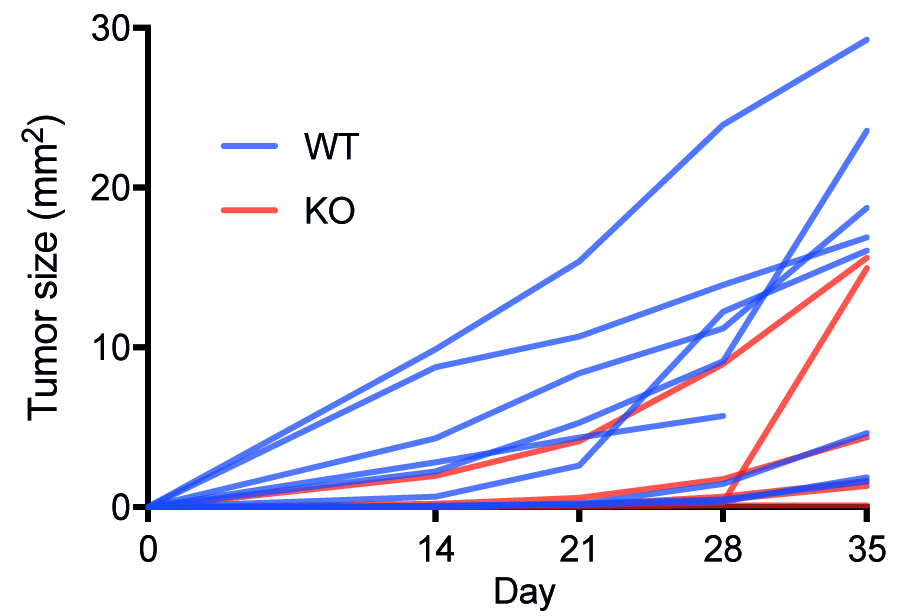

Supplement: Supplemental Figure 2 — Individual tumor area as measured via epifluorescence. n = 9 mice until day 28, and n = 8 mice on day 35 (U-2932-tdt, blue); n = 6 mice (U-2932-tdt-KO, red). [file Image_2.TIF]
